# The relationship between lower limb maximal and explosive strength and change of direction ability: Comparison of basketball and tennis players, and long-distance runners

**DOI:** 10.1371/journal.pone.0256347

**Published:** 2021-08-18

**Authors:** Žiga Kozinc, Darjan Smajla, Nejc Šarabon

**Affiliations:** 1 University of Primorska, Faculty of Health Sciences, Izola, Slovenia; 2 University of Primorska, Andrej Marušič Institute, Koper, Slovenia; 3 InnoRenew CoE, Human Health Department, Izola, Slovenia; 4 S2P, Science to Practice, Ltd., Laboratory for Motor Control and Motor Behavior, Ljubljana, Slovenia; University of Innsbruck, AUSTRIA

## Abstract

Change-of-direction (CoD) ability is an important determinant of athletic performance. Muscle strength is among the most important determinants of CoD ability. However, previous studies investigating the relationship between CoD ability and muscle strength focused mostly on flexor and extensor muscle groups, or used multi-joint exercises, such as jumps, squats or mid-thigh pull. The purpose of the present study was to investigate the relationship between CoD ability and strength of ankle, knee, hip and trunk maximal and explosive strength. The participants (n = 327), consisting of male and female basketball players, tennis players and long-distance runners completed isometric strength assessments and CoD testing (90° and 180° turn tests). The times of both CoD tests were associated with muscle strength (peak torques and the rate of torque development variables), with correlation coefficients being mostly weak to moderate (r = 0.2–0.6). Strength variables explained 33%, 62% and 48% of the variance in the 90° turn task, and 42%, 36% and 59% of the variance in the 180° turn task, in basketball players, long-distance runners and tennis players, respectively. Hip and trunk muscle strength variables were the most prevalent in the regression models, especially hip adduction and abduction strength. Our results suggest that the strength of several lower limb muscles, in particular of the hip abductors and adductors, and trunk muscles, but also hip rotators, extensors and flexors, as well as knee and ankle flexors and extensors should be considered when aiming to improve CoD performance.

## Introduction

Change-of-direction (CoD) ability is an important determinant of athletic performance within several sports [1, 2], with various CoD maneuvers being routinely executed during gameplay and training. For instance, soccer players were reported to perform up to 800 cutting movements per game and basketball players show a very high frequency of lateral movements (up to 450 per game) [3]. It is not surprising that a considerable amount of research has been dedicated to investigating interventions to improve CoD ability. Recent systematic reviews have shown a high potential of plyometric training for improving CoD ability [4, 5], and a recent study showed significant improvements in CoD ability (effect size = 1.35) independent of the predominant direction of force vectors in training (horizontal vs. vertical) and mode of training (training for maximal strength vs. explosive strength) [6]. It is clear that muscular strength, particularly of the lower limbs, is of paramount importance for CoD performance [6–8]. However, because previous studies failed to perform a comprehensive assessment of maximal and explosive strength, less is known about the importance of individual joints or muscle groups.

Spiteri et al. [9] reported high correlations (r = -0.79 to -0.89) between CoD ability (T-test and 505 test) and performance on several isometric, concentric and eccentric multi-joint strength and power tests (squat, mid-thigh pull, vertical jumps) in elite female basketball athletes. Slightly lower correlations (r = -0.57 to -0.62) were reported between the modified 505 CoD test and isometric mid-thigh pull variables (peak force and force impulses) in male collegiate athletes [10]. Similar correlations (r = -0.60 to -0.67) were reported between the 505 test and eccentric (60°/s) knee flexion and extension strength for a sample of female soccer players [11]. In male and female national Olympic team handball players, T-test performance was highly correlated with squat jump height (r = 0.66), countermovement jump height (r = 0.82) and mean propulsive power during loaded jump squat (r = 0.51) [7]. Another study, conducted on a sample of university students, reported that running speed was the most important determinant of 505 CoD test performance (i.e. 57% of explained variance in the linear regression model), while the additional predictive value of eccentric knee flexion strength increased the explained variance to 67%. [12]. In sum, it is clear that lower limb strength and power, as well as linear sprint ability are related to CoD performance. However, previous studies have been mostly conducted on relatively small sample sizes (n = 10–40), and strength assessments included sagittal plane movement patterns (e.g. vertical jumping test, mid-thigh pull test, squat jumps). Moreover, previous studies included either multi-joint exercises or a limited number of single-joint assessments. Of note, the function of the trunk has also been implicated to affect CoD performance [13]. To the best of our knowledge, no study to date has prior examined the relationship between CoD performance and a comprehensive assessment of lower limb and trunk strength.

The purpose of this study was to investigate the relationship between CoD ability and maximal strength (peak joint torques) as well as explosive strength (rate of torque development (RTD)) of the trunk extensors, flexors and lateral flexors, hip flexors, extensors, internal and external rotators, abductors and adductors, knee flexors and extensors, and ankle flexors and extensors. We investigated a larger sample of athletes from basketball and tennis, as CoD performance is an important determinant of performance in these sports [3, 14], and a group of long-distance runners for comparison. In accordance with previous reports showing high correlations between CoD performance and muscle strength and power, we hypothesized that the outcome variables related to maximal and explosive strength would be able to explain at least 50% of the variance in CoD performance. We also hypothesized that basketball and tennis players would exhibit superior CoD performance compared to long-distance runners, and that strength would play a lesser role in CoD performance in long-distance runners.

## Methods

### Participants and study design

The study sample was comprised of 327 professional athletes (age: 18.6 ± 8.1 years; body mass = 71.2 ± 13.1 kg; body height = 179.2 ± 10.5 cm), specifically 163 basketball players (57 females), 102 tennis players (42 females) and 49 long-distance runners (19 females). Details regarding the age, body mass, body height, years of training and weekly training frequency are provided in Table 1. Participants were recruited through national sports associations and regional or local sports clubs. Participants with lower leg injuries in the past 6 months and any neurological or musculoskeletal injuries or were excluded from the study. Participants were thoroughly informed about the experimental procedures and written informed consent was required prior to commencing the study. For underage participants, their parents or legal guardians were also thoroughly informed and signed the consent on their behalf. The experiment was approved by Republic of Slovenia National Medical Ethics Committee (approval no. 0120-99/2018/5) and was conducted in accordance with the latest revision of the Declaration of Helsinki.

**Table 1:** Basic participant data. The numbers in the brackets represent sample sizes within each subgroup.

	Sex		Sport		
	Male (203)	Female (120)	Basketball (164)	Running (49)	Tennis (104)
Age (years)^#^	18.2 ± 7.8	19.5 ± 8.7	16.8 ± 1.4	32.8 ± 10.2	16.9 ± 8.6
Body Height (cm) ^$^^#^	182.2 ± 9.7	171.1 ± 8.1	183.9 ± 9.9	176.1 ± 10.2	172.1 ± 10.9
Body Mass (kg) ^$^^#^	73.66 ± 12.7	64.9 ± 11.5	76.8 ±13.1	71.5 ± 11.1	62.9 ± 12.6
Regular training (years) ^$^^#^	8.1 ± 4.4	7.2 ± 3.3	6.6 ± 2.5	10.2 ± 7.6	8.7 ± 3.8
Weekly training sessions ^$^^#^	6.1 ± 2.4	5.6 ± 2.4	5.9 ± 1.8	4.6 ± 2.4	6.3 ± 3.1

$- significant effect of sex

#—significant effect of sport.

### Study design, tasks and procedures

The measurements were conducted within a single session, lasting approximately 3 hours. The participants were asked to refrain from strenuous physical activities at least 48 hours prior to testing. The study was designed as a cross-sectional experiment, wherein the participants performed CoD tasks and isometric strength assessments for the trunk extensors, flexors and lateral flexors, hip flexors, extensors, internal and external rotators, abductors and adductors, knee flexors and extensors, and ankle flexors and extensors. The order or the measurement sections (with CoD tasks and each joint being tested on a different section) was counter-balanced between the participants. The order of the tasks within the section (e.g. extensions and flexion for ankle strength assessment) was randomized. Before the measurements, the participants performed a 20-min warmup (10 min of light running on an indoor track, 5 minutes of dynamic stretching and 5 min of low-intensity resistance exercises).

#### Change of direction assessment

**Fig 1:**
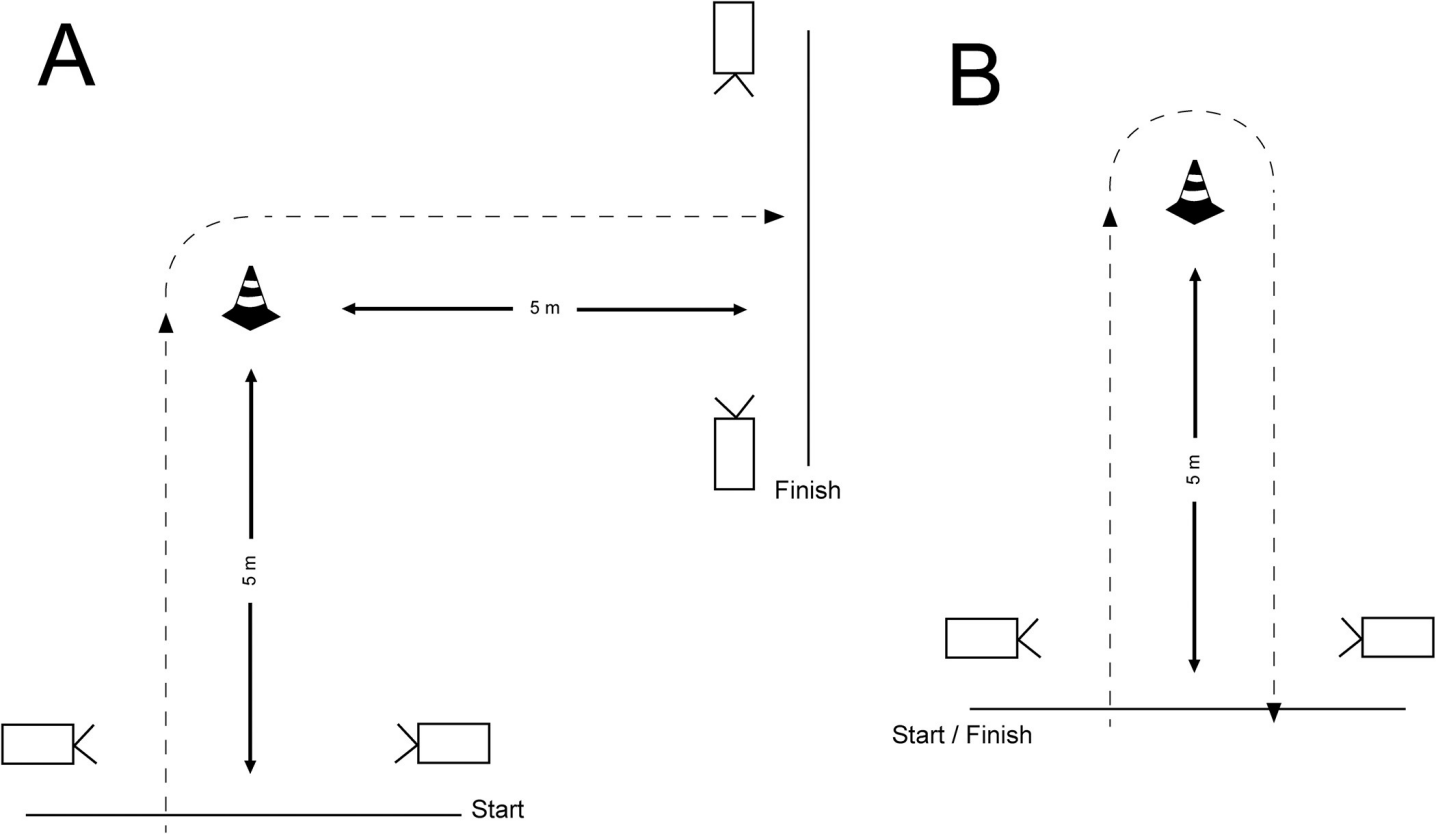
Representation of CoD_90_ (A) and CoD_180_ tasks.

CoD tests were performed on a tartan floor in a gym. Single-beam photocell timing gates (Brower Timing Systems, Draper, UT, USA) were positioned as shown in Fig 1. The tests were conducted in accordance to previous studies [15–17]. The timing gates were placed at about hip height and the starting line was 0.5 m behind the first timing gate to prevent early triggering. Before the test trials, the participants completed 2 familiarization trials (for each task) at 50 and 75% of their maximal effort. Then, 3 repetitions were recorded for each side (left and right) and each task (CoD_90_ and CoD_180_), with rest periods of 1 min between repetitions and 3 min between tasks, for a total of 12 trials. For the CoD_90_, the participants were instructed to start at their own convenience, sprint around the cone, make a 90° turn on to either left or right, and sprint through the finish line (Fig 1A). For the CoD_180_, the participants sprinted around the cone and back to the first timing gate (10 m in total, Fig 1B). Loud verbal encouragement was provided at all times to ensure maximum effort. For each task, the best time was taken for each side (left and right turn). In our previous studies using identical set-up, very consistent CoD_90_ and CoD_180_ times were observed across repetitions [15].

#### Strength assessments

All strength assessments were conducted using isometric dynamometers from the same manufacturer (S2P, Science to Practice, Ljubljana, Slovenia) with embedded force sensors (model 1-Z6FC3/200kg or model PW2DC3/72KG, HBM, Darmstadt, Germany). For each task, three repetitions were performed with 1 min break between repetitions The break between the measurement sections, defined by joint, was at least five minutes. In order to quantify peak torque and RTD, the participants were instructed to push ‘‘as fast and as hard as possible” [18] and sustain the maximal effort for ~3 s, while loud verbal encouragement was given. The same trials were used for analysis of peak torque and RTD outcome measurements. Different procedures were used for trunk strength assessment as the participants were instructed to gradually increase the torque and sustain the maximal effort for ~ 3 s. Consequently, RTD was not assessed for the trunk, as RTD measurements with this dynamometer have been observed by the researchers to have questionable validity and reliability.

**Fig 2:**
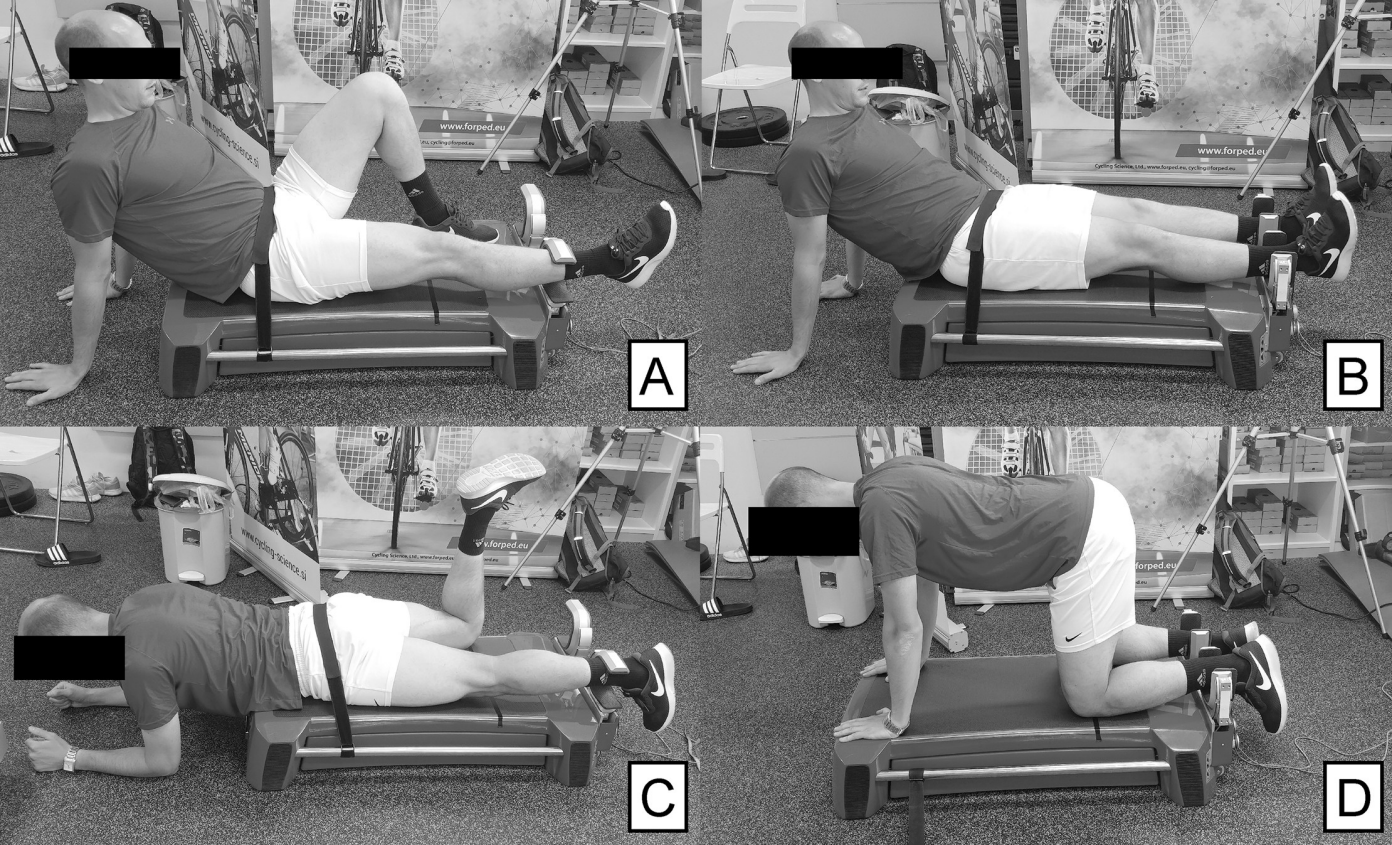
Assessments for the hip joint were all performed on MuscleBoard dynamometer. (A–hip flexion, B–hip abduction and adduction, C–hip extension, D–hip external and internal rotation).

The strength of the hip extensors, flexors, internal and external rotators, abductors and adductors, was assessed with the MuscleBoard dynamometer, in accordance with previous studies [19, 20]. The dynamometer has been shown to provide highly reliable peak torque outcomes for the hip muscle groups [21]. The dynamometer comprises of two U-shaped braces with single point load cells. The braces may be rotated to accommodate the desired task. For the assessment of hip flexors (FLX) (Fig 2A), abductors (ABD) and adductors (ADD) (Fig 2B), the participants were seated on the dynamometer and maintained hip flexion at ~ 45° [21], while supporting themselves by placing the forearms on the floor next to the dynamometer with shoulders flexed to approximately 90°. The leg was positioned against the load cell on the level just above the malleoli. For the assessment of hip extensors (EXT) (Fig 2C), the participants were in a prone position and supported themselves by placing the forearms on the floor next to the dynamometer. For the assessment of hip external and internal rotators, (ER and IR, respectively), the participants were in the prone four-point position, supporting themselves on the hands and knees, with the hip and the knees bent at 90° (Fig 2D). For all measurements in the prone and supine position, a tight fixation across the pelvis was provided. Measurements of ABD, ADD, ER and IR were performed bilaterally, while EXT and FLX were performed unilaterally in random order.

**Fig 3:**
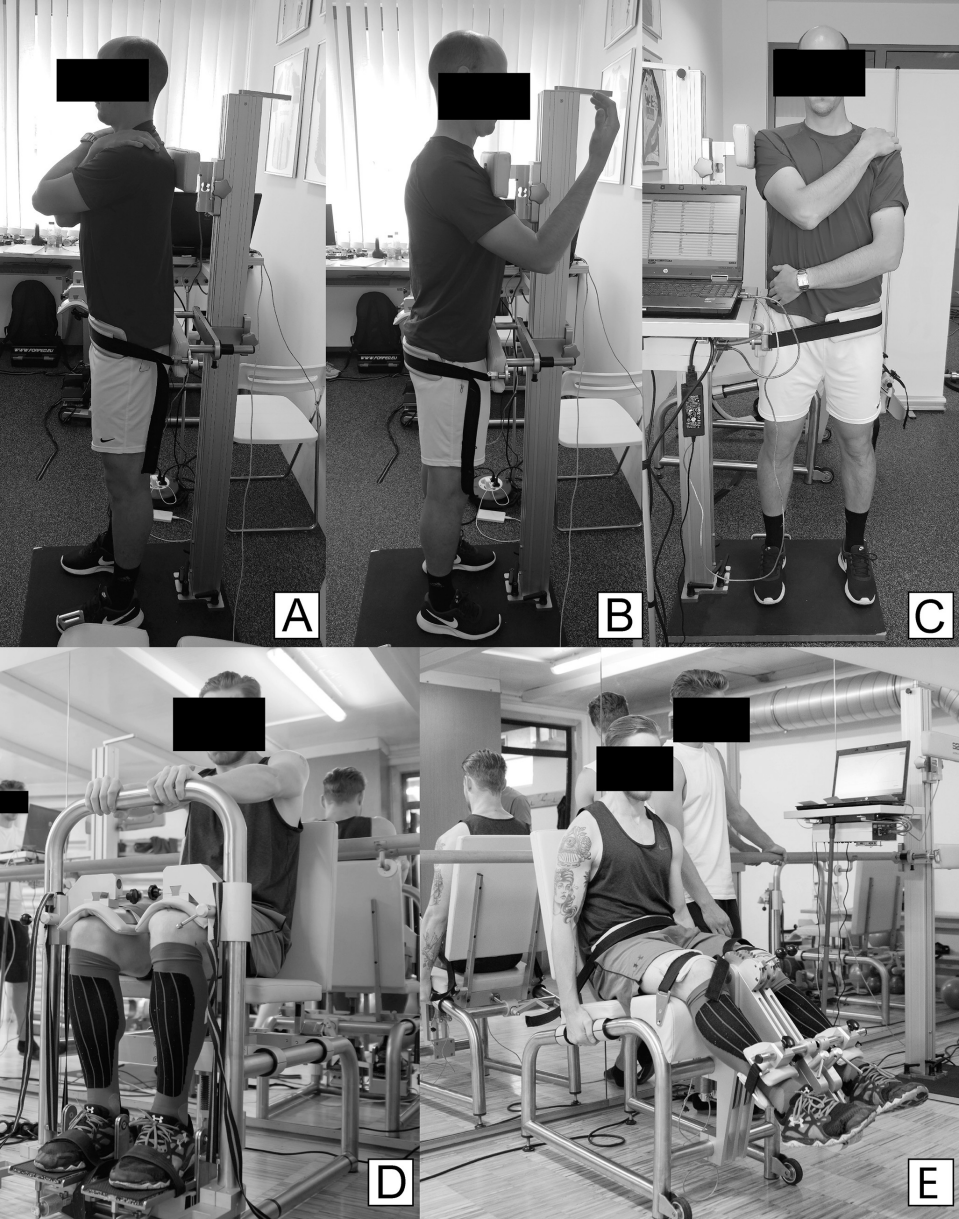
Trunk, knee and ankle strength assessments (A–trunk extension, B–trunk flexion, C–trunk lateral flexion, D–ankle flexion and extension, E–knee flexion and extension).

The trunk dynamometer design and measurement procedures have been described in detail elsewhere [22]. For the assessment of the trunk extensors, flexors and lateral flexors strength (EXT, FLX and LFLX, Fig 3A–3C), the participants were positioned next to the dynamometer and were fixated across the pelvis. The force sensor was placed at the level of spina scapulae and was kept constant for all the tasks The arms were positioned on the shoulder or were hanging alongside the body (depending on the task) to avoid any contribution to force recordings.

For the assessment of the ankle flexors and extensors strength (Fig 3D), the participant’s shins were tightly secured within the dynamometer, and the feet were placed on a rigid plate mounted on a torque sensor. The axis of the dynamometer was aligned with the medial malleolus and the ankle was in a neutral position (90° in the sagittal plane). The foot was tightly fixated against the plate with a strap. The whole leg was also tightly secured with additional braces placed on the thigh just over the knee. For the assessment of the knee flexor and extensor strength (Fig 3E), the participant was seated in the dynamometer and fixated across the pelvis and the lower portion of the thighs. The axis of the dynamometer was aligned with the lateral femoral condyle and the knee angle was set to 60° (0° = full extension). Very high reliability of the knee dynamometer was demonstrated in our previous study [23].

### Data processing and outcome measures

All torque signals were recorded at a sampling rate of 1000 Hz, except for the hip tasks (450 Hz). The signals were further automatically processed (arithmetic mean filter, 5 ms window) by the manufacturer’s software (Analysis and Reporting Software, S2P, Ljubljana, Slovenia). For all of the strength assessments, the peak torque value during each maximal voluntary contraction trial was quantified as the maximal 1-s mean value of the trial. The onset of torque rise was automatically detected at the instant at which the baseline signal exceeded the 3% of the peak torque. In addition, offline manual inspection for potential software errors was done. The instance of onset of torque rise, as detected by the software, was shown by a marker, which could be moved manually if needed (~ 3.0% of cases). Next, the RFD was calculated as Δ torque / Δ time for 0–50 (RTD_50_) and 0–100 ms (RTD_100_) time intervals after the onset of the contraction. As noted in the previous section, RTD could not be considered for trunk strength assessments. All peak torque and RTD outcomes were normalized to body mass. For all of the outcome variables, the best repetition (the highest peak torque or RTD value) was used for further statistical analyses.

### Statistical analysis

Statistical analyses were performed in SPSS (version 25.0, SPSS Inc., Chicago, USA). Descriptive statistics are reported as mean ± standard deviation. The normality of the data distribution was assured with Shapiro-Wilk tests. The within-session reliability for all outcome measurements was assessed with two-way random, single measures intra-class correlation coefficient (ICC) for absolute agreement. The reliability according to ICC was interpreted as poor (< 0.5), moderate (0.5–0.75), good (0.75–0.9) and excellent (>0.9) [24]. Furthermore, typical errors were calculated according to the guidelines by Hopkins [25], and expressed as coefficient of variation (CV). The CV < 10% was considered to reflect acceptable reliability. The differences between sexes and sports groups were assessed with 2-way univariate analysis of variance, with effect sizes expressed as eta-squared (η^2^) and interpreted as small (~0.01), medium (~0.06) and large (> 0.14) [26]. Correlations among outcome variables were assessed with Pearson’s correlation coefficients and interpreted as negligible (< 0.1), weak (0.1–0.4), moderate (0.4–0.7), strong (0.7–0.9) and very strong (> 0.9). Multiple linear stepwise regressions were done with CoD_90_ and CoD_180_ as dependent variables and all peak torque and RTD variables as candidate predictors. The successive predictors were included in the model if they statistically significantly (p < 0.05) contributed to the proportion of explained variance in CoD_90_ and CoD_180_. Durbin-Watson statistics and collinearity diagnostic tests were performed. We conservatively set the thresholds for the presence of collinearity at ≤ 0.3 for tolerance and ≥ 3 for variance inflation factor [27]. For all analyses, the threshold for statistical significance was set at p < 0.05.

## Results

The normality of the data distribution was confirmed with Shapiro-Wilk tests (p ≥ 0.092). There was a large age difference between sports (p < 0.001; η^2^ = 0.4), mostly because the runners were notably older than basketball and tennis players (see Table 1). Body height and body mass was also significantly different between the sexes and among the sports (p < 0.001; η^2^ = 0.22–0.24 for body height; 0.11–0.18 for body mass). Similarly, there were sex and sport differences for years of regular training (p = 0.014–0.020; η^2^ = 0.18–0.28) and weekly training sessions (p = 0.001–0.040; η^2^ = 0.14–0.55) (see Table 1 for descriptive statistics).

### Reliability

The times of both CoD tasks showed good or excellent relative reliability (ICC = 0.88–0.97 across sports) and acceptable absolute reliability (CV = 2.10–3.25%). All peak torque outcomes showed excellent relative reliability (ICC = 0.93–0.98) and acceptable absolute reliability (CV = 1.68–4.89%).

RTD outcomes for the knee showed moderate to good relative reliability (ICC = 0.59–0.85) and unacceptable absolute reliability (CV = 10.43–26.71%), with the exception of EXT outcomes in tennis players (CV = 8.19–9.34%). RTD outcomes for the ankle showed moderate to good relative reliability (ICC = 0.70–0.90) and acceptable absolute reliability (CV = 6.50–9.91%), with the exception of EXT RTD_50_ in basketball players (CV = 11.30%). Relative reliability for hip RTD outcomes was moderate to good in basketball players (ICC = 0.54–0.76), runners (ICC = 0.58–0.87) and tennis players (ICC = 0.65–0.87). Absolute reliability for all hip RTD outcomes was unacceptable in basketball players (CV = 14.64–26.15%) and tennis players (CV = 10.74–18.23%). In runners, the absolute reliability was acceptable for RTD_100_ outcomes (CV = 8.03–9.85%) and unacceptable for RTD_50_ outcomes (CV = 14.63–19.55%). The reliability analysis is included in Table 2. In the S1 File, an extended reliability analysis with confidence intervals for ICC and CV is also provided.

**Table 2:** Reliability scores for all outcome measures.

Sport	Basketball		Running		Tennis	
Outcome variable	ICC	CV	ICC	CV	ICC	CV
CoD90 (s)	0.91	3.25	0.97	2.52	0.88	2.48
CoD180 (s)	0.91	2.31	0.94	2.50	0.89	2.10
Knee EXT—Peak torque	0.94	3.50	0.95	3.52	0.96	3.63
Knee EXT—RTD_50_	0.64	17.84	0.79	10.34	0.84	9.34
Knee EXT–RTD_100_	0.65	16.87	0.76	10.97	0.85	8.19
Knee FLX—Peak torque	0.93	3.71	0.97	2.88	0.95	3.98
Knee FLX—RTD_50_	0.59	26.71	0.73	23.01	0.68	19.12
Knee FLX–RTD_100_	0.65	19.99	0.76	19.67	0.74	16.34
Ankle EXT—Peak torque	0.96	3.01	0.97	2.80	0.97	2.60
Ankle EXT—RTD_50_	0.71	11.30	0.84	8.91	0.85	8.41
Ankle EXT—RTD_100_	0.77	9.52	0.86	7.54	0.90	6.50
Ankle FLX—Peak torque	0.96	2.31	0.98	1.96	0.93	3.78
Ankle FLX—RTD_50_	0.70	9.21	0.78	9.91	0.88	7.76
Ankle FLX—RTD_100_	0.79	7.02	0.87	6.68	0.91	5.75
Hip ABD—Peak torque	0.97	2.21	0.98	1.80	0.98	2.25
Hip ABD—RTD_50_	0.68	20.18	0.74	17.14	0.76	17.87
Hip ABD—RTD_100_	0.61	15.96	0.85	8.03	0.82	12.83
Hip ADD—Peak torque	0.94	3.32	0.98	2.23	0.96	3.51
Hip ADD—RTD_50_	0.54	26.15	0.67	19.55	0.81	16.91
Hip ADD—RTD_100_	0.62	17.45	0.87	8.52	0.87	10.74
Hip ER- Peak torque	0.96	2.63	0.98	1.68	0.97	3.00
Hip ER- RTD_50_	0.63	24.83	0.69	15.10	0.77	15.61
Hip ER—RTD_100_	0.71	14.69	0.81	9.25	0.79	12.73
Hip IR—Peak torque	0.94	3.29	0.97	2.45	0.98	2.87
Hip IR—RTD_50_	0.67	19.24	0.68	14.63	0.79	15.22
Hip IR—RTD_100_	0.61	16.94	0.79	9.85	0.87	11.66
Hip EXT—Peak torque	0.94	4.24	0.94	4.06	0.95	4.14
Hip EXT—RTD_50_	0.72	21.48	0.63	18.32	0.83	15.77
Hip EXT—RTD_100_	0.65	14.64	0.86	9.78	0.85	12.32
Hip FLX—Peak torque	0.95	3.38	0.95	2.92	0.96	3.06
Hip FLX—RTD_50_	0.67	24.56	0.58	16.09	0.65	18.23
Hip FLX—RTD_100_	0.76	16.50	0.85	9.16	0.77	13.92
Trunk EXT—Peak Torque	0.95	4.89	0.97	3.78	0.98	3.37
Trunk FLX—Peak Torque	0.96	3.90	0.97	3.26	0.96	4.14
Trunk LFLX—Peak Torque	0.94	4.39	0.98	2.37	0.96	3.98

EXT–extension; FLX–flexion; ABD–abduction; ADD–adduction; ER–external rotation; IR–internal rotation; RTD–rate of torque development; ICC–intra-class correlation coefficient; CV–coefficient of variance.

### Change of direction performance and strength

There were statistically significant main effects of sport group for CoD_90_ (p < 0.001; η^2^ = 0.18) and CoD_180_ (p < 0.001; η^2^ = 0.14). All pair-wise post-hoc tests showed statistically significant differences between sports for CoD_90_ (p = 0.001–0.014). In CoD_180_, tennis players and runners showed similar results (p = 1.000), while the remainder of the pairwise comparisons (basketball players compared to runners or tennis players) were statistically significant (p < 0.001) (see Table 2 for descriptive statistics). Males had shorter CoD_90_ (mean difference: 0.25 s) and CoD_180_ (mean difference: 0.23 s) times (p < 0.001) compared to females (See S1 File for details on descriptive statistics according to sex).

The main effect of sport was statistically significant (all p < 0.001; η^2^ = 0.05–0.53) for all trunk, knee and ankle measures (peak torques and RTDs), except Knee FLX peak torque (p = 0.080) (see Table 3 for descriptive statistics). For the hip, statistically significant differences between sports were found for all peak torque variables (p = 0.001–0.002; η^2^ = 0.02–0.13), except IR (p = 0.261). In terms of RTD at the hip, only ADD RTD_100_ showed main effect of sport (p = 0.024; η^2^ = 0.03). The differences between sexes were statistically significant for all variables, except Hip IR RTD_50_ (p = 0.155) and Knee FLX RTD_50_ (p = 0.263) (See S1 File for descriptive statistics according to sex).

**Table 3:** Descriptive statistics for all outcome variables.

Sport	Basketball		Running		Tennis		Effect of sport	
Outcome variable	Mean	SD	Mean	SD	Mean	SD	Sig.	η^2^
CoD90 (s)	2.47	0.15	2.67	0.38	2.56	0.17	<0.001	0.18
CoD180 (s)	2.97	0.17	3.03	0.27	3.07	0.19	<0.001	0.13
Knee EXT—Peak torque	2.40	0.51	2.96	0.84	3.38	0.79	<0.001	0.24
Knee EXT—RTD_50_	8.75	3.55	16.91	6.75	22.51	6.56	<0.001	0.53
Knee EXT–RTD_100_	10.01	3.78	15.35	5.77	19.14	5.29	<0.001	0.37
Knee FLX—Peak torque	1.57	0.31	1.60	0.43	1.62	0.38	0.080	0.02
Knee FLX—RTD_50_	5.06	2.38	3.78	2.29	5.63	2.32	<0.001	0.11
Knee FLX–RTD_100_	5.86	2.33	4.93	2.59	6.76	2.60	<0.001	0.13
Ankle EXT—Peak torque	3.64	0.88	3.44	0.84	3.63	0.82	<0.001	0.12
Ankle EXT—RTD_50_	12.87	4.03	12.33	4.33	13.10	3.91	<0.001	0.06
Ankle EXT—RTD_100_	16.21	4.85	15.14	4.66	16.18	4.53	<0.001	0.07
Ankle FLX—Peak torque	1.07	0.29	1.05	0.15	0.96	0.17	<0.001	0.27
Ankle FLX—RTD_50_	6.13	1.57	5.90	1.48	5.49	1.49	<0.001	0.21
Ankle FLX—RTD_100_	5.82	1.61	5.43	1.17	5.06	1.16	<0.001	0.25
Hip ABD—Peak torque	1.91	0.32	1.78	0.34	1.88	0.36	<0.001	0.06
Hip ABD—RTD_50_	5.07	2.98	5.65	3.18	5.58	3.32	0.073	0.02
Hip ABD—RTD_100_	8.21	3.22	7.71	2.42	7.95	3.50	0.796	0.00
Hip ADD—Peak torque	1.88	0.39	1.86	0.44	1.91	0.41	<0.001	0.13
Hip ADD—RTD_50_	5.12	3.66	5.19	3.25	5.96	3.87	0.353	0.01
Hip ADD—RTD_100_	8.71	4.08	7.96	3.11	9.10	3.87	0.024	0.02
Hip ER- Peak torque	1.70	0.31	1.58	0.28	1.69	0.34	<0.001	0.13
Hip ER- RTD_50_	4.88	3.41	4.39	1.93	4.41	2.19	0.470	0.01
Hip ER—RTD_100_	6.99	2.87	6.39	2.23	6.48	2.52	0.251	0.01
Hip IR—Peak torque	1.99	0.39	1.91	0.43	2.01	0.48	0.261	0.01
Hip IR—RTD_50_	4.30	2.44	4.70	1.96	4.51	2.30	0.123	0.01
Hip IR—RTD_100_	6.71	3.01	6.84	2.24	6.51	3.08	0.371	0.01
Hip EXT—Peak torque	2.21	0.49	2.13	0.53	2.16	0.56	<0.001	0.07
Hip EXT—RTD_50_	5.01	2.91	5.65	3.15	5.54	2.99	0.519	0.01
Hip EXT—RTD_100_	8.31	3.56	8.58	3.80	8.22	3.75	0.261	0.01
Hip FLX—Peak torque	1.89	0.36	1.80	0.31	1.90	0.38	0.002	0.04
Hip FLX—RTD_50_	4.20	2.72	4.48	2.71	4.50	2.58	0.783	0.00
Hip FLX—RTD_100_	6.53	2.93	6.47	2.58	6.66	2.75	0.186	0.01
Trunk EXT—Peak Torque	3.46	1.05	3.68	1.17	3.27	1.06	<0.001	0.13
Trunk FLX—Peak Torque	2.62	0.74	2.67	0.79	2.51	0.77	<0.001	0.10
Trunk LFLX—Peak Torque	2.44	0.70	2.42	0.60	2.41	0.73	<0.001	0.06

All peak torque values are in Nm/kg and all RTD values are in Nm/kg/s. EXT–extension; FLX–flexion; ABD–abduction; ADD–adduction; ER–external rotation; IR–internal rotation; RTD–rate of torque development; SD–standard deviation.

### Association between change of direction performance and strength

Table 4 contains correlation coefficients between CoD performance (i.e. CoD_90_ and CoD_180_ times) and peak torque as well as RTD outcomes. All statistically significant correlation coefficients were negative, which means that higher peak torques or RTDs were associated with shorter CoD times. The correlation coefficients were mostly weak or moderate. While almost all strength outcome variables were statistically significantly correlated with CoD performance in basketball and tennis, there very fewer significant correlations in runners, in particular for the ankle and the ankle joint (1/10 coefficients). In tennis players, the coefficients tended to be higher for CoD_180_ compared to CoD_90_, while no clear trend was observed in basketball and running. Of note, trunk strength outcomes showed several of the highest correlations with CoD performance (four coefficients > 0.6).

**Table 4:** Associations (correlation coefficients) between CoD performance and strength outcome variables.

		Knee						Ankle						Trunk		
		EXT			FLX			EXT			FLX			EXT	FLX	LFLX
		PT	RTD_50_	RTD_100_	PT	RTD_50_	RTD_100_	PT	RTD_50_	RTD_100_	PT	RTD_50_	RTD_100_	PT	PT	PT
Basketball	CoD_90_	-.39**	-.26**	-.28**	-.41**	-.16*	-.21**	-.42**	-.38**	-.44**	-.22**	-.32**	-.29**	-.42**	-.39**	-.42**
	CoD_180_	-.33**	-.23**	-.24**	-.46**	-0.14	-.23**	-.38**	-.37**	-.38**	-.29**	-.41**	-.39**	-.36**	-.32**	-.38**
Running	CoD_90_	-.36*	-0.22	-0.24	-.48**	-0.01	-0.08	-.30*	-0.19	-.30*	-0.12	-0.04	-0.17	-.62**	-.68**	-.67v
	CoD_180_	-.45****	-0.16	-0.22	-.48**	-0.15	-0.24	-0.22	-0.05	-0.09	-0.17	-0.05	-0.05	-.36**	-0.25	-0.18
Tennis	CoD_90_	-.38**	-.38**	-.41**	-.50**	-.31**	-.47**	-.23*	-.24*	-.29**	-.39**	-.36**	-.37**	-.39**	-.48**	-.47**
	CoD_180_	-.52**	-.49**	-.51**	-.61**	-.40**	-.53**	-.28**	-.32**	-.37**	-.48**	-.39**	-.47**	-.49**	-.61**	-.56**
		Hip														
		ABD			ADD			ER			IR			EXT		
		PT	RTD_50_	RTD_100_	PT	RTD_50_	RTD_100_	PT	RTD_50_	RTD_100_	PT	RTD_50_	RTD_100_	PT	RTD_50_	RTD_100_
Basketball	CoD_90_	-.38**	-.16*	-.29**	-.47**	-.29**	-.40**	-.40**	-0.14	-.23**	-.27**	-.15*	-.23**	-.36**	-.18*	-.24**
	CoD_180_	-.33**	-.23**	-.30**	-.52**	-.28**	-.41**	-.47**	-.19*	-.27**	-.26**	-.23**	-.27**	-.18*	-.16*	-.20**
Running	CoD_90_	-.34*	0.26	-0.01	-.54**	-0.15	-.34*	-.50**	-0.03	-0.14	-.38**	0.05	-0.08	-.52**	-0.15	-.28*
	CoD_180_	-.37**	-.40**	-.31*	-.37**	-.38**	-.30*	-0.20	-0.23	-0.19	-0.08	-0.12	-0.04	-.31*	-0.25	-0.22
Tennis	CoD_90_	-.45**	-0.15	-.23*	-.56**	-.39**	-.41**	-.48**	-0.15	-.32**	-.49**	-.31**	-.33**	-.47**	-.25*	-.27**
	CoD_180_	-.57**	-.32**	-.37**	-.63**	-.41**	-.46**	-.54**	-.24*	-.43**	-.51**	-.40**	-.44**	-.38**	-.29**	-.34**

* p < 0.05

** p < 0.01; PT–peak torque; EXT–extension; FLX–flexion; ABD–abduction; ADD–adduction; ER–external rotation; IR–internal rotation; RTD–rate of torque development; For clarity and outlay of the table, Hip FLX are omitted. The correlation coefficients were very similar to Hip EXT, or slightly smaller (difference in r = 0.01–0.06).

### Regression analyses

No collinearity was detected in any of the regression analyses (all tolerance values > 0.3, all VIF < 3.0). The details with all regression models are available in S1 File. Strength variables explained 33%, 62% and 48% of the variance in CoD_90_ in basketball players, long-distance runners and tennis players, respectively. In the basketball players model (R^2^ = 0.33), mostly peak torques (Hip ADD, Hip EXT, Trunk EXT, Hip FLX) were included, in addition to Ankle EXT RTD_100_. In the model for runners (R^2^ = 0.62), three peak torque variables were included (Trunk EXT, Trunk FLX, Hip ABD). Finally, the model for tennis players (R^2^ = 0.48), included 3 peak torque values (Hip ADD, Hip IR, Knee EXT), in addition to Knee FLEX RTD_100_ and Hip IR RTD_50_.

Furthermore, strength variables explained 42%, 36% and 59% of the variance in CoD_180_ in basketball players, long-distance runners and tennis players, respectively. In the basketball model (R^2^ = 0.42), two peak torques (Hip ADD, Knee FLX) and two RTD variables (Ankle FLEX RTD100, Knee EXT RTD_50_) were included. The model for runners (R^2^ = 0.36) included Knee FLX peak torque, Hip ABD RTD_50_ and Hip IR RTD_100_. Finally, the model for tennis players (R^2^ = 0.59), included 4 peak torque values (Hip ADD, Hip ABD, Trunk LFLEX, Ankle FLX) in addition to Knee EXT RTD_50_.

## Discussion

The purpose of this study was to investigate the relationship between CoD performance and maximal and explosive strength of the trunk, hip, knee and ankle muscle groups in basketball and tennis players and long-distance runners. Basketball players showed the best CoD_90_ performance, followed by tennis players and runners. Less differences were observed for CoD_180_, with basketball players showing slightly better performance than tennis players and runners. The correlation coefficients between CoD tasks and peak torque and RTD values were mostly small or moderate (r = 0.25–0.65). Strength variables explained 33%, 62% and 48 in CoD_90_, and 42%, 36% and 59% of the variance in CoD_180_ in basketball players, long-distance runners and tennis players, respectively. Hip and trunk variables were the most prevalent in the regression models.

Muscular strength and power are important determinants of CoD ability [1, 28]. This is not surprising, given that CoD tasks require quick accelerations and decelerations of the body, which is in turn underpinned by maximal force and power producing capacities. Accordingly, previous studies have reported high correlations between CoD performance and various measures of lower limb strength and power [7, 9, 11, 28]. Moreover, it has been reported that strength training can be an effective method for improving CoD ability [5, 29]. However, some studies reported no such effect. For instance, de Hoyo et al. [30] observed no effects of either of their 3 strength exercise-based in-season interventions (squat training, resisted sprint training or plyometric training) on CoD ability in young elite soccer players, despite clear increases in jumping ability and sprint performance. Similarly, a 10-week training program, consisting of a combination of multi-joint and single-joint lower limb resistance exercises, did not elicit improvement in CoD ability in female soccer players [31]. Therefore, further research is warranted to clarify this issue and optimize resistance training programs for the purpose of improvements in CoD performance.

The present study showed that the strength in the frontal (Hip ABD and ADD, Trunk LFLX) (r = 0.16–0.67) and transverse plane (Hip ER and IR) (r = 0.15–0.54) show similar correlations with CoD performance as those obtained in the sagittal plane (ankle, knee and hip FLX and EXT) (r = 0.16–0.61). Moreover, outcome variables pertaining to trunk strength showed some of the highest associations with CoD ability. This suggests that strengthening of the muscles that generate force predominantly in frontal and transverse plane might be just as important as the strengthening of the muscles that act predominantly in the sagittal plane. It could be that the lack of effects of resistance exercise programs on CoD performance in some studies [30, 31] was observed because EXT and FLX strength training was overemphasized at the expense of other muscle groups. Accordingly, based on the increases in CoD test times following trunk muscle fatigue protocol, Roth et al. [32] have suggested that trunk strength should not be neglected when aiming at improving CoD ability, although leg fatiguing did induce even larger negative effects. Moreover, it was shown that lateral pelvic tilt range during the change of direction cutting maneuver was associated (r = 0.55) with worse performance times. It could be that coordinated stabilization of the body, driven primarily by hip and trunk muscles, is important for optimizing sagittal plane movements within CoD maneuvers. On the other hand, higher trunk range of motion (relative to pelvis) during CoD task was reported to be beneficial for male sport athletes [13]. Due to the equivocal results, further studies are needed to reveal how important trunk strength is for performing CoD tasks, and whether trunk stability or range of motion are beneficial or detrimental to CoD performance. Evidence from interventional studies show that targeted trunk resistance exercise can favorably modify lower limb biomechanics during drop jumps and single-leg squats [33], as well as side-step cutting [34]. While these findings highlight the potential of trunk resistance exercise for injury prevention, the effects on CoD performance is less clear. Prieske et al. [35] reported no effect of trunk-focused resistance training on T-test times in elite youth soccer players. Similarly, Sever & Zorba [36] reported no effects of core exercises on 505 test and ‘‘arrowhead” agility test performance in semi-professional soccer players, and Ozmen & Aydogmus [37] observed no effects of similar training on Illinois test performance in young badminton players. On the other hand, Bashir et al. [38] observed improvements in t-test performance after trunk strengthening program in young tennis players. In sum, it seems that trunk strengthening could be used to improve CoD ability in some cases, however, future interventional studies are needed before more comprehensive recommendations can be given.

Previous studies have mostly used correlational analysis when studying determinants of CoD performance, which can limit the interpretation of the importance of individual factors. One of the previous studies using regression analysis reported that 57% of the variance in 505 CoD test time could be explained with linear sprint times, while knee FLX strength contributed an additional 10% [12]. This finding is expected, as linear sprinting represents a considerable portion of CoD tasks. Nevertheless, the regression models in the present studies, based solely on maximal and explosive strength measures, were able to explain a considerable (33–62%) amount of variance in two CoD tasks, again pointing to high importance of strength for CoD performance. The differences in regression models among sport groups are more difficult to explain. Higher between-participant variability in CoD performance in runners could have contributed to the high proportion in explained variance (62%) in CoD_90_ task. On the other hand, as the difficulty of the task increased (CoD_180_), factors other than strength could become more prominent (e.g. coordination, familiarity with similar tests), reducing the explained variance to 36%. Moreover, eccentric strength seems to be a particularly important determinant of performance of sharp CoD maneuvers [11, 39], while CoDs at lower angles are highly dependent on linear sprinting capability [39]. In basketball and tennis, which are both characterized by CoD elements, strength of the lower limbs and trunk is suggested to play an important role in CoD performance. Further studies should investigate interventions including exercises targeting hip ABD/ADD and ER/IR as well as the trunk to confirm their importance for CoD performance.

### Limitations

The sample of long-distance runners was notably older than the samples of basketball and tennis players, and were also less familiar to performing CoD tasks. Therefore, the effect of age and task familiarity could have confounded our results. The inter-repetition reliability was similar among groups, which suggests that task familiarity is not a huge issue. It has been shown that CoD ability increases rapidly in early adolescence [40]. On the other hand, there was no correlation (r = 0.03) between CoD performance and age within a sample of female basketball players, aged 18 to 32 years (the low and high end range correspond approximately to younger (basketball, tennis) and older (runners) participants in our study). Another limitation of this study was the inclusion of isometric strength assessments. Based on previous studies [9, 12], the addition of eccentric and/or concentric outcome variables could have improved the proportion of explained variance in regression models. In particular, eccentric strength could be of higher importance for CoD performance [41], especially for the deceleration phase of the CoD maneuvers [42, 43]. On the other hand, isometric strength is more easily assessed in practice, as most isokinetic dynamometers enable isometric measurements, but not vice versa. Moreover, peak joint torques may be also assessed by hand-held dynamometry with decent reliability [44]. Finally, we did not consider direction-specificity in this study. It has been implied that inter-limb asymmetries in lower limb strength are related to asymmetries in CoD performance [20]. However, it has been recently stressed that asymmetries in CoD ability should not be calculated based on raw test times. Rather, the time needed to cover the distance of the CoD test in a single straight run should be first subtracted from CoD times to isolate the true CoD asymmetry [45–47]. Because 10 m sprints (the total distance of CoD_90_ and CoD_180_ tasks in our study was 10 m) were not assessed in this study, we did not study the aspect of asymmetries in CoD and its relationship with maximal and explosive strength. In addition, the CoD_180_ test involved turning around a cone, which assesses maneuverability and CoD ability at the same time. The results of a more isolated CoD ability test, such as 505 test, could exhibit different associations with peak torque and RTD outcomes. As mentioned above, CoD ability can also be further isolated by calculation CoD deficit [47], which should be considered for future studies. It could also be argued that modified 505 test (not involving 10 m acceleration phase as the traditional version) is more similar to our CoD_180_ test than traditional 505 test. The time to complete the modified 505 test was shown to be correlated (r = -0.57 to -0.62) with isometric mid-thigh pull variables (peak force and force impulses) in male collegiate athletes. Finally, we used the same trials to quantify peak torque and RTD variables. While this was done to minimize fatigue, it has to be stressed that the validity and the reliability of the RTD is expected to be lower when it is derived from the same trial as peak torque [18]. Indeed, we observed high within-participant variations in many of the RTD outcomes in the present study. Future studies should use separate trials for RTD assessment if possible, with an emphasis on instruction to produce fast pulses with high peak force, but without sustaining the contraction [18]. The suboptimal reliability is probably the most important limitation of this study. At the same time, it has to be stressed that the reliability scores we provide reflects inter-repetition reliability and not test-retest reliability. We used the best repetition (the highest peak torque or RTD value) for all outcome measures for further statistical analyses, which discarded the suboptimal repetitions (e.g. participants not giving maximal effort), which are the likely culprit for poor reliability.

## Conclusions

The novel finding of this study is that the strength of Hip ABD, ADD, IR and ER, as well as trunk muscles, show moderate positive associations with CoD performance. Thus, the results of this study imply that the strength of lower limb muscles other than FLX and EXT groups (notably hip ADD, ABD, IR and ER) and trunk muscles should also be considered when aiming to improve CoD performance. Therefore, strengthening of all lower limb muscles, as well as the trunk muscles should be considered for this purpose, however, further interventional studies are needed to determine the optimal resistance exercise programs to improve CoD performance.

## Supporting information

S1 File Detailed statistical results. (XLSX)
